# Trends in the Use and Outcomes of Mechanical Ventilation among Patients Hospitalized with Acute Exacerbations of COPD in Spain, 2001 to 2015

**DOI:** 10.3390/jcm8101621

**Published:** 2019-10-04

**Authors:** Javier de Miguel-Diez, Rodrigo Jiménez-García, Valentin Hernández-Barrera, Luis Puente-Maestu, Walther Iván Girón-Matute, José M. de Miguel-Yanes, Manuel Méndez-Bailón, Rosa Villanueva-Orbaiz, Romana Albaladejo-Vicente, Ana López-de-Andrés

**Affiliations:** 1 Respiratory Department, Hospital General Universitario Gregorio Marañón, Facultad de Medicina, Universidad Complutense de Madrid, Instituto de Investigación Sanitaria Gregorio Marañón (IiSGM), 28007 Madrid, Spain; javier.miguel@salud.madrid.org (J.d.M.-D.); luis.puente@salud.madrid.org (L.P.-M.); walter_giron2@hotmail.com (W.I.G.-M.); 2 Department of Public Health & Maternal and Child Health, Faculty of Medicine, Universidad Complutense de Madrid, 28040 Madrid, Spain; mrvillan@med.ucm.es (R.V.-O.); ralbadal@med.ucm.es (R.A.-V.); 3 Preventive Medicine and Public Health Teaching and Research Unit, Health Sciences Faculty, Rey Juan Carlos University, Alcorcón, 28922 Madrid, Spain; valentin.hernandez@urjc.es (V.H.-B.); ana.lopez@urjc.es (A.L.-d.-A.); 4 Internal Medicine Department, Hospital General Universitario Gregorio Marañón, Facultad de Medicina, Universidad Complutense de Madrid, Instituto de Investigación Sanitaria Gregorio Marañón (IiSGM), 28007 Madrid, Spain; josemaria.demiguel@salud.madrid.org; 5 Internal Medicine Department, Hospital Clínico San Carlos, 28040 Madrid, Spain; manuel.mendez@salud.madrid.org

**Keywords:** chronic obstructive pulmonary disease, hospitalization, invasive mechanical ventilation, non-invasive mechanical ventilation, trends

## Abstract

(1) Background: We examine trends (2001–2015) in the use of non-invasive ventilation (NIV) and invasive mechanical ventilation (IMV) among patients hospitalized for acute exacerbation of chronic obstructive pulmonary disease (AE-COPD). (2) Methods: Observational retrospective epidemiological study, using the Spanish National Hospital Discharge Database. (3) Results: We included 1,431,935 hospitalizations (aged ≥40 years) with an AE-COPD. NIV use increased significantly, from 1.82% in 2001–2003 to 8.52% in 2013–2015, while IMV utilization decreased significantly, from 1.39% in 2001–2003 to 0.67% in 2013–2015. The use of NIV + invasive mechanical ventilation (IMV) rose significantly over time (from 0.17% to 0.42%). Despite the worsening of clinical profile of patients, length of stay decreased significantly over time in all types of ventilation. Patients who received only IMV had the highest in-hospital mortality (IHM) (32.63%). IHM decreased significantly in patients with NIV + IMV, but it remained stable in those receiving isolated NIV and isolated IMV. Factors associated with use of any type of ventilatory support included female sex, lower age, and higher comorbidity. (4) Conclusions: We found an increase in NIV use and a decline in IMV utilization to treat AE-COPD among hospitalized patients. The IHM decreased significantly over time in patients who received NIV + IMV, but it remained stable in patients who received NIV or IMV in isolation.

## 1. Introduction

Chronic obstructive pulmonary disease (COPD) is characterized by chronic airflow limitation that is not completely reversible and is often progressive [1]. It is a leading cause of morbidity and mortality worldwide and its prevalence is increasing [2,3]. 

The natural course of COPD is characterized by the occurrence of exacerbations (usually two to three per year), which are acute events characterized by a worsening of COPD symptoms, often leading to additional treatments, emergency room visits, or hospitalizations [4]. Furthermore, they result in a substantial burden on patients and healthcare systems [5], being a major contributor to the economic costs of COPD [6]. 

In hospitalized patients for an acute exacerbation of COPD (AE-COPD), usual care includes bronchodilators, corticosteroids, antibiotics, supplemental oxygen and, in severe cases, ventilator support, either with non-invasive ventilation (NIV) or invasive mechanical ventilation (IMV) [7]. The main characteristic of NIV, in comparison with IMV, is that the pressure is applied through a mask, thus avoiding endotracheal intubation [8].

Currently, the role of NIV in the treatment of acute respiratory failure during AE-COPD is well established, with overwhelming evidence of reducing the need for IMV and improved survival in carefully selected patients [9,10,11,12,13,14]. It is thought that this benefit may be mediated, at least in part, through the prevention of complications related with IMV use, including ventilator-associated pneumonia and barotrauma [15]. 

Although the indications for NIV have expanded over the last few decades [16], there are limited data on the epidemiology of COPD exacerbations necessitating mechanical ventilation in daily clinical practice [17,18,19]. Furthermore, recent studies have demonstrated that application of this type of ventilator support in real-life settings remains suboptimal [20]. 

Using data from the population based Spanish National Hospital Discharge Database (SNHDD), we examined fifteen-year trends (2001–2015) in the use of NIV and IMV among patients hospitalized for an AE-COPD and assessed the influence of patient factors on receipt of NIV and IMV.

## 2. Materials and Methods

### 2.1. Design, Setting, and Participants

We conducted an observational retrospective epidemiological study. Our data source was the SNHDD. The SNHDD contains de-identified clinical and resource utilization data of over 95% of the hospital discharges per year in Spain. Details of the database are described elsewhere [21].

Briefly, for each hospital discharge demographic characteristics, up to 14 diagnoses and up to 20 procedures are included in the database. International Classification of Diseases, Ninth Revision, Clinical Modification (ICD-9-CM) is used for coding. Also, hospital outcome variables such as length of hospital stay (LOHS) and in hospital mortality (IHM) are collected by the SNHDD. 

For study purpose we included hospitalizations of patients ≥40 years of age. We considered to suffer an AE-COPD those with a principal discharge diagnosis of COPD (ICD-9-CM codes: 491.21, 492.22, 491.8, 491.9, 492.8, 496) or a principal diagnosis of acute respiratory failure (ICD-9-CM codes: 518.81, 518.82, 518.84) or pneumonia (ICD-9-CM codes 480 to 487) paired with a secondary diagnosis of COPD who were discharged between 01/01/2001 and 31/12/2015 as described Stefan et al. [17]. We excluded all hospitalizations with an ICD-9-CM code of surgery as described Metha et al. [22] in any procedure field in the SNHDD, as these patients could receive ventilator support for the surgery and for their medical conditions.

We considered a patient to have received NIV or IMV during the admission if there was an ICD-9-CM procedure code for NIV (93.90, 93.91) or IMV (96.70, 96.71, 96.72) in any procedure field. 

We defined three cohorts of patients, those who received only NIV, only IMV and NIV + IMV. The register does not allow establishing the temporal sequence of treatments, and the NIV + IMV group did thus encompass both NIV succeeded by IMV and IMV succeeded by NIV.

### 2.2. Study Variables

For each hospital admission we recorded covariates such as demographic information (age and sex), diagnosed comorbidities and hospital variables such as LOHS and IHM. IHM was defined by the proportion of patients who died during admission for each year of study.

To assess the burden of comorbidity, all those conditions included in the Charlson Comorbidity Index (CCI) coded in any diagnosis position in the discharge report were identified [23]. Points were assigned to individual diseases as described by Toft-Petersen et al. [18] and grouped in three with 1, 2 or 3+ points respectively as per definition all patients received one point for having COPD. We also identified pneumonia as a comorbidity if patients had pneumonia as their principal diagnosis or a principal diagnosis of acute respiratory failure with a secondary diagnosis of pneumonia, or a principal diagnosis of sepsis (ICD-9-CM 038.x, 995.91, 995.92, or 785.52) with a secondary diagnosis of pneumonia. Finally, we also analyzed readmission rate, discharge location and services the patients were discharged from. A patient is considered a readmission if he had been discharged from the same hospital in the previous month. According to the SNHDD the discharge location included five possible options: died in the hospital, home, other hospital, nursing home and other. We provide data for the following discharging hospital services: internal medicine, respiratory medicine, geriatric medicine, intensive care medicine, other medical service, emergency room and other.

### 2.3. Statistical Methods

We calculated summary statistics for all variables using frequencies and proportions for categorical data and means (standard deviations) for continuous variables. We compared differences in continuous variables using the Student t test, or ANOVA as appropriate. Categorical variables were compared using Chi-square tests.

We used multinomial logistic regression to examine relative changes over time in the use and outcomes of NIV, IMV and NIV + IMV while controlling for several patients and hospital characteristics. 

Variables included in the models were those that yielded a significant association in the bivariate analysis. Odds ratios with 95% confidence intervals are shown.

All statistical analyses were performed using Stata Software 11.0 (StataCorp LP, College Station, Texas, USA).

### 2.4. Ethical Aspects

Retrospective use of de-identified register data does not require ethical approval or informed consent according to Spanish legislation. The Spanish Ministry of Health (SMH) provided the database and gave us permission to use the data after we signed an engagement in which we legally promise to (i) under no circumstances export the entire database or make partial exports that could allow the generation of the same through aggregation or identification of natural persons or reporting units, and (ii) destroy the file or data provided and all the copies made of it once elapsed the period of time for which the data is required. 

## 3. Results

A total of 1,431,935 hospitalizations of patients aged 40 years or older with an AE-COPD in Spain (2001–2015) were included. 

An AE-COPD was identified more frequently among men (84.68%) than women. The percentage of males affected decreased significantly (*p* < 0.001) over time (84.77% in 2001–2003 vs. 83.05% in 2013–2015). The mean age at admission was 74.63 years (SD 10.27 years) and 27.85% of hospitalizations had CCI ≥3. We found that 20.1% of hospitalizations had a concomitant diagnosis of pneumonia. The mean age, CCI and concomitant pneumonia diagnosis increased significantly over time (Table 1).

Mean LOHS decreased significantly from 2001–2003 to 2013–2015 (9.57 days vs. 8.04 days). Over the fifteen years, IHM was 6.87%. IHM decreased from 6.88% in 2001–2003 to 6.61 in 2013–2015 (*p* < 0.01) (Table 1).

### 3.1. Trends in the Use of NIV and IMV between 2001 and 2015 and Time Trends of Ventilation Use from 2001 to 2015

#### 3.1.1. Trends in the Use of NIV and IMV between 2001 and 2015

According to the SNHDD, 88,229 patients with an AE-COPD had received ventilator support in Spain from 2001 to 2015. Of them, 77.99% received only NIV, 16.93% only IMV and 5.08% both procedures.

**Figure 1:**
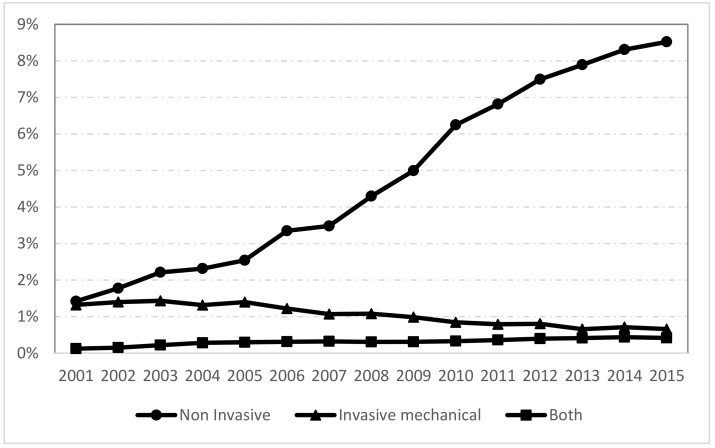
Trends in mechanical ventilation among patients hospitalized with acute exacerbations of chronic obstructive pulmonary disease (COPD) in Spain, 2001 to 2015.

As can be seen in Figure 1, the use of NIV in patients with AE-COPD increased significantly from 1.42% in 2001 to 8.52% in 2015, however the use of IMV decreased over time (1.32% in 2001 vs. 0.66% in 2015; *p* < 0.001). The use of NIV + IMV rose significantly from 0.12% to 0.42% over time.

Table 2 describes the characteristics of hospital admission in patients with AE-COPD according to the type of ventilation received.

For all the types of ventilator support the male predominance is constant; however, the gender differences are reducing significantly over time.

According to age, those receiving NIV (71.55 years) were the oldest, followed closely by IMV (69.12 years) and those who required NIV + IMV (68.49 years). Mean age increased significantly over time in those who underwent NIV but decreased in those who required IMV and NIV + IMV.

Patients who required NIV had the highest value of CCI ≥3 (28.33%) with similar values for IMV (27.79%) and NIV + IMV (27.69%). For all types of ventilation, values of CCI ≥3 increased significantly over the study period (all *p* < 0.001).

Pneumonia was most frequently diagnosed in patients with IMV (24.18%), followed by those who underwent NIV + IMV (20.27%) and NIV (11.92%). For all types of ventilation, prevalence of pneumonia increased significantly over time (all *p* < 0.001).

The highest mean LOHS is found in those who received NIV + MIV (17.22 days) and the lowest for NIV (10.07 days). LOHS decreased significantly in all types of ventilation from 2001–2003 to 2013–2015.

Patients who received only IMV had the highest IHM (32.63%) followed by those with NIV + IMV (27.09%) and NIV (12.12%). The IHM decreased significantly from 30.79% to 24.18% in patients with NIV + IMV from 2001–203 to 2013–2015 (*p* < 0.001), however in patients with NIV and IMV the IHM remained stable with figures around 12% for NIV and 32% for IMV.

Shown in Supplementary Table S1 are the readmission rates and discharge location of admissions for exacerbation of COPD from 2001 to 2015 in Spain according to form of ventilation. The readmission rates are around 17% for those who did not require ventilation (NV) and those with NIV with figures significantly lower (12% approx.) for those with IMV or NIV + IMV. The readmission rate has increased significantly overtime for the NV and the NIV groups. 

Besides the type of ventilation or if the patient had not received any ventilatory support, the most frequent discharge location was “home”, followed by “other hospital” that represents 2.07% among the NV patients, 1.97% for NIV, 7.46% for IMV and 6.01% for NIV + IMV. Discharges to nursing homes are very infrequent, representing 2.14% for the NIV + IMV and smaller proportions for the other groups analyzed.

The discharge units for exacerbation of COPD from 2001 to 2015 in Spain according to form of ventilation are shown in Supplementary Table S2. When patients with exacerbation of COPD have not received ventilation, the most frequent service that discharges the patient is Internal Medicine (50.96%) with a significant increment from 45.87% in 2004–2006 to 52.56% in 2013–2015 (*p* < 0.001). The second service among NV patients is Respiratory with 32.56% of patients discharged.

For all the groups of patients with any form of ventilatory support Respiratory Service has the highest discharge proportion, with figures of 59.34% for NIV, 36.83% for IMV and 51.35% for NIV + IMV. In all cases the second service is Internal Medicine. Intensive Care Medicine service discharges 24.4% and 19.62% of patients who underwent IMV and NIV + IMV respectively.

#### 3.1.2. Time Trends of Ventilation Use from 2001 to 2015

As can be seen in Table 3, after multinomial logistic regression over the 15-year study, compared to admissions without ventilation, the probability of receiving NIV and NIV + IMV increased significantly over time (OR 1.147; 95% CI 1.145–1.149 and OR 1.08; 95% CI 1.07–1.09, respectively); however, IMV decreased significantly over time (OR 0.953; 95% CI 0.950–0.957).

Factors associated with a greater probability of receiving NIV vs. not being ventilated included female sex, lower age, and higher CCI whereas suffering pneumonia reduced the probability. For MIV and NIV + MIV the factors that increased the probability of receiving these types of ventilation are the same as for NIV. However, for these two types, unlike those with NIV, those with pneumonia had higher risk of being ventilated.

## 4. Discussion

In this large observational study, we evaluated real-life trends in use of mechanical ventilation in patients hospitalized with AE-COPD in Spain from 2001 to 2015. We found a significant increase in NIV use, accompanied by a concomitant reduction in the use of IMV. On the other hand, the proportion of patients requiring treatment with NIV + IMV during the hospitalization increased over time.

Like us, other authors have reported that more NIV use is associated with less IMV utilization [24,25]. Several factors can justify these trends. In first place, previous studies have demonstrated that NIV is efficacious in reducing the need for IMV and IHM in selected patients with acute respiratory failure caused by COPD [9,10,12,26,27]. Consequently, NIV is now a standard component of the management of these patients and is included in the most recent international guidelines [28,29]. On the other hand, unlike IMV, NIV can be implemented outside the Intensive Care Units (ICU) [24]. Some hospitals have even created special units, commonly located next to the UCI, to facilitate NIV use [30].

The proportion of patients requiring combined treatment with NIV + IMV during the hospitalization increased over time in the present study. However, we do not know which of the two types of ventilator support was performed in the first place. It is difficult to compare these findings with those published previously because of differences in study design. Chandra et al. [24] reported that the proportion of patients requiring transition from NIV to IMV during the hospitalization remained stable from 1998 to 2008. These patients experienced the longest hospitalization and the highest IHM, worsening mortality in this group over time. By contrast, the highest IHM in our study was reported in patients who received only IMV and the mortality improved over time in those who received treatment with NIV + IMV. It is possible that, in a significant percentage of these patients, NIV has been an alternative to ventilate patients who initially fail weaning attempts from IMV, since previous studies have demonstrated the benefits of this strategy [31].

We observed a change in the clinical profile of patients over time. Although the male predominance was constant for all the types of ventilator support used, the gender differences were reducing significantly over time. Furthermore, mean age increased significantly over the study period in those who underwent NIV but decreased in those who required IMV and treatment with NIV + IMV. In the same way Stefan et al. [17], in a large observational study of hospitalizations on COPD patient in the United States, found that the use of NIV over a period of 10 years increased faster in older patients relative to the youngest, while the utilization of IMV decreased more among the oldest. Results of previous studies reporting the good success rate in older patients with NIV use could justify the trends described [32,33]. On the other hand, comorbidity increased significantly over the study period for patients receiving any type of ventilation in our study. Comorbidity burden has been identified as an important determinant of whether a patient receives ventilator support and influences the annual rate of change in the use of different ventilation strategies [17]. Concomitant pneumonia was an important determinant of the type of mechanical ventilation used in our study. For all types of ventilation, pneumonia increased significantly over time. It was most frequently diagnosed in patients with IMV, followed with those who underwent NIV + IMV and NIV. Other authors have described that, compared with patients without pneumonia, those with AE-COPD and pneumonia are more likely to lead to any ventilation, especially IMV initiation, and to experience NIV failure [15,17]. 

Despite the worsening of clinical profile of our patients over time, the LOHS decreased significantly in all types of ventilation. IMH also decreased, but only in patients who did not receive mechanical ventilation or in those who received treatment with NIV+IMV, remaining stable in groups treated with isolated NIV or isolated IMV. Trethewey et al [34] also found that IHM remained stable and LOHS decreased over time, despite greater comorbidity and more severe acute respiratory failure in COPD patients treated for the first time with ward-based NIV. It is possible that increasing experience with NIV allow to maintain the IHM stable despite an increase in the severity of illness. Similar results were reported by Toft-Petersen et al. [18], who reported that IHM remained stable among patients receiving NIV only despite this type of ventilator support had been initiated in increasingly older patients. By contrast, other authors have found decreasing mortality rates among patients receiving NIV [24,25]. It is unclear whether changes in mortality are related with variations in the patterns of mechanical ventilation. In any case, these results indicate that the management of patients hospitalized by EA-COPD has been improving over time. 

Factors associated with a greater probability of receiving any type of mechanical ventilation in our population study included female sex, lower age, and higher CCI whereas suffering pneumonia reduced the probability for NIV. Other authors have reported that the presence of pneumonia, as well as associated complications, comorbid conditions and older age are factors associated with NIV failure [35,36].

The strengths of our study are the large number of patients evaluated, increasing the generalizability of our findings, and the high reliability of the SNHDD [21]. In addition, we examined trends over a long period, which provides a perspective on the ventilator management of COPD patients during the study time. Last, we identified factors associated with the use of mechanical ventilation over time and possible changes in these factors over the perspective of an extended period of time. 

However, our study has noteworthy limitations. In the first place, we used ICD-9 codes to identify patients with AE-COPD, which could be subject to misclassification [37]. Another limitation to our study is the lack of information on baseline pulmonary function, severity of the exacerbation, and appropriate use of pharmacological therapies (e.g., steroids and bronchodilators). In addition, we did not have information about the do-not-intubate status of COPD patients or its preferences for end-of-life care. We have not included data on type of hospital because this variable was not provided to us by the Spanish Ministry of Health (for confidentiality reasons), so it is not possible to know the size or if the hospital was a university hospital. Regarding geographical analysis, it is not possible because Spain is divided into 17 regions and 51 provinces. The population of the regions range from 8.5 million to only 350,000 inhabitants and for provinces from 6.5 million to under 100,000 inhabitants therefore making comparisons not reliable. Finally, the SNHDD only includes information of the patient during the hospital admission, but not afterwards, so it is not possible to assess the mortality or the evolution of these patients after discharge. 

## 5. Conclusions

Our study provides important information about trends in mechanical ventilation use for AE-COPD and associated outcomes. We found an increase in NIV use and a decline in IMV utilization to treat AE-COPD among hospitalized patients in Spain from 2001 to 2015. Factors associated with use of any type of ventilator support included female sex, lower age, and higher CCI. The IHM decreased significantly over time in patients who received NIV + IMV, but it remains stable in patients who received NIV or IMV in isolation. Additional research is needed to identify factors associated with appropriate use of NIV and/or IMV and maximize benefits of mechanical ventilation during AE-COPD.

## Figures and Tables

**Table 1:** Characteristics of admissions for exacerbation of COPD from 2001 to 2015 in Spain.

		2001–2003	2004–2006	2007–2009	2010–2012	2013–2015	Total	*p*
Sex, *n* (%)	Male	227,159 (84.77)	244,122 (85.62)	262,861 (85.39)	243,934 (84.49)	234,464 (83.05)	1,212,540 (84.68)	<0.001
	Female	40,815 (15.23)	41,007 (14.38)	44,968 (14.61)	44,769 (15.51)	47,836 (16.95)	219,395 (15.32)	
Age	Mean (SD)	73.6 (9.85)	74.24 (9.96)	74.72 (10.25)	75.24 (10.46)	75.26 (10.71)	74.63 (10.27)	<0.001
Age groups in years, *n* (%)	40–64	43,592 (16.27)	45,053 (15.8)	49,955 (16.23)	45,997 (15.93)	46,486 (16.47)	231,083 (16.14)	<0.001
	65–74	89,012 (33.22)	86,832 (30.45)	83,077 (26.99)	71,382(24.73)	70,830 (25.09)	401,133 (28.01)	
	75–84	103,098 (38.47)	115,080 (40.36)	125,418 (40.74)	118,041 (40.89)	109,007 (38.61)	570,644 (39.85)	
	≥85	32,272 (12.04)	38,164 (13.38)	49 379 (16.04)	53,283 (18.46)	55,977 (19.83)	229,075 (16)	
Charlson comorbidity index, *n* (%)	1	144,016 (53.74)	137,056 (48.07)	135 531 (44.03)	114,458 (39.65)	105,536 (37.38)	636,597 (44.46)	<0.001
	2	72,471 (27.04)	80,072 (28.08)	86,132 (27.98)	80,587 (27.91)	77,274 (27.37)	396,536 (27.69)	
	≥3	51,487 (19.21)	68,001 (23.85)	86,166 (27.99)	93,658 (32.44)	99,490 (35.24)	398,802 (27.85)	
Pneumonia, *n* (%)	Yes	47,426 (17.7)	53,678 (18.83)	58,416 (18.98)	61,497 (21.3)	66,808 (23.67)	287,825 (20.1)	<0.001
Ventilation type, *n* (%)	NV	258,932 (96.63)	272,759 (95.66)	290,577 (94.4)	265,526 (91.97)	255,912 (90.65)	1,343,706 (93.84)	<0.001
	NIV	4881 (1.82)	7776 (2.73)	13,072 (4.25)	19,789 (6.85)	23,293 (8.25)	68,811 (4.81)	
	IMV	3716 (1.39)	3747 (1.31)	3216 (1.04)	2346 (0.81)	1904 (0.67)	14,929 (1.04)	
	NIV + IMV	445 (0.17)	847 (0.3)	964 (0.31)	1042 (0.36)	1191 (0.42)	4489 (0.31)	
Length of hospital stay	Mean (sd)	9.57 (8.32)	9.25 (7.94)	8.88 (7.98)	8.33 (7.63)	8.04 (6.89)	8.81 (7.79)	<0.001
In-hospital mortality, *n* (%)	Yes	18,436 (6.88)	20,170 (7.07)	21,289 (6.92)	19,866 (6.88)	18,667 (6.61)	98,428 (6.87)	<0.001

NV: no ventilation. NIV: non-invasive ventilation. IMV: invasive ventilation.

**Table 2:** Characteristics of admissions for exacerbation of COPD from 2001 to 2015 in Spain in according to form of ventilation.

	Type	2001–2003	2004–2006	2007–2009	2010–2012	2013–2015	Total
Male Sex, *n* (%)	NV*	219,352 (84.71)	233,526 (85.62)	248,336 (85.46)	224,806 (84.66)	213,329 (83.36)	1,139,349 (84.79)
	NIV *	4198 (86.01)	6611 (85.02)	10,959 (83.84)	16,328 (82.51)	18,656 (80.09)	56,752 (82.48)
	IMV *	3230 (86.92)	3235 (86.34)	2738 (85.14)	1940 (82.69)	1543 (81.04)	12,686 (84.98)
	NIV + IMV *	379 (85.17)	750 (88.55)	828 (85.89)	860 (82.53)	936 (78.59)	3753 (83.6)
Age, mean (sd)	NV *	73.75 (9.82)	74.43 (9.91)	74.97 (10.21)	75.56 (10.4)	75.63 (10.65)	74.87 (10.22)
	NIV *	69.76 (9.71)	70.31 (10.2)	70.98 (10.21)	72.13 (10.49)	72.16 (10.6)	71.55 (10.42)
	IMV *	68.73 (9.47)	69.44 (9.5)	69.75 (9.88)	68.67 (10.2)	68.69 (10.08)	69.12 (9.77)
	NIV + IMV *	68.34 (9.16)	69.37 (9.4)	68.29 (9.79)	68.49 (9.75)	68.07 (10.02)	68.49 (9.71)
CCI 1, *n* (%)	NV *	139,286 (53.79)	131,316 (48.14)	128,147 (44.1)	105,453 (39.71)	95,592 (37.35)	599,794 (44.64)
CCI 2, *n* (%)		69,919 (27)	76,253 (27.96)	80,898 (27.84)	73,505 (27.68)	69,421 (27.13)	369,996 (27.54)
CCI ≥3, *n* (%)		49,727 (19.2)	65,190 (23.9)	81,532 (28.06)	86,568 (32.6)	90,899 (35.52)	373,916 (27.83)
CCI 1, *n* (%)	NIV *	2684 (54.99)	3716 (47.79)	5660 (43.3)	7653 (38.67)	8703 (37.36)	28,416 (41.3)
CCI 2, *n* (%)		1331 (27.27)	2438 (31.35)	4023 (30.78)	6117 (30.91)	6992 (30.02)	20,901 (30.37)
CCI ≥3, *n* (%)		866 (17.74)	1622 (20.86)	3389 (25.93)	6019 (30.42)	7598 (32.62)	19,494 (28.33)
CCI 1, *n* (%)	IMV *	1844 (49.62)	1665 (44.44)	1305 (40.58)	940 (40.07)	759 (39.86)	6513 (43.63)
CCI 2, *n* (%)		1074 (28.9)	1103 (29.44)	923 (28.7)	653 (27.83)	514 (27)	4267 (28.58)
CCI ≥3, *n* (%)		798 (21.47)	979 (26.13)	988 (30.72)	753 (32.1)	631 (33.14)	4149 (27.79)
CCI 1, *n* (%)	NIV + IMV *	202(45.39)	359 (42.38)	419 (43.46)	412 (39.54)	482 (40.47)	1874 (41.75)
CCI 2, *n* (%)		147 (33.03)	278 (32.82)	288 (29.88)	312 (29.94)	347 (29.14)	1372 (30.56)
CCI ≥3, *n* (%)		96 (21.57)	210 (24.79)	257 (26.66)	318 (30.52)	362 (30.39)	1243 (27.69)
Pneumonia *n* (%)	NV *	45,902 (17.73)	51,789 (18.99)	56,277 (19.37)	58,429 (22.01)	62,703 (24.5)	275,100 (20.47)
	NIV *	507 (10.39)	808 (10.39)	1272 (9.73)	2276 (11.5)	3342 (14.35)	8205 (11.92)
	IMV *	927 (24.95)	928 (24.77)	697 (21.67)	576 (24.55)	482 (25.32)	3610 (24.18)
	NIV + IMV *	90 (20.22)	153 (18.06)	170 (17.63)	216 (20.73)	281 (23.59)	910 (20.27)
LOHS, mean (sd)	NV *	9.4 (8.11)	9.05 (7.7)	8.66 (7.66)	8.08 (7.37)	7.77 (6.57)	8.6 (7.53)
	NIV *	12.03 (9.7)	11.52 (8.96)	11.22 (10.02)	10.42 (8.71)	10.07 (8.19)	10.69 (8.93)
	IMV *	16.67 (13.97)	16.26 (13.59)	15.96 (14.52)	15.07 (12.04)	14.45 (12.09)	15.88 (13.5)
	NIV + IMV *	19.94 (14.75)	19.39 (14.54)	18.34 (14.37)	18.87 (14.3)	17.22 (12.86)	18.52 (14.06)
IHM, *n* (%)	NV *	16,513 (6.38)	17,756 (6.51)	18,378 (6.32)	16,470 (6.2)	14,883 (5.82)	84,000 (6.25)
	NIV	559 (11.45)	932 (11.99)	1579 (12.08)	2375 (12)	2895 (12.43)	8340 (12.12)
	IMV	1227 (33.02)	1264 (33.73)	1046 (32.52)	734 (31.29)	601 (31.57)	4872 (32.63)
	NIV + IMV *	137 (30.79)	218 (25.74)	286 (29.67)	287 (27.54)	288 (24.18)	1216 (27.09)

* *p*-trend < 0.05. NV: no ventilation. NIV: non-invasive ventilation. IMV: invasive ventilation. CCI: Charlson comorbidity index. LOHS: length of hospital stay. IHM: in-hospital mortality.

**Table 3:** Trends over time (2001–2015) in the type of ventilation used adjusted by patient characteristics. Results of multinomial logistic regression models.

	NIV/No Ventilation	IMV/No Ventilation	NIV + IMV/No Ventilation
	OR (95%CI)	OR (95%CI)	OR (95%CI)
Year	1.147 (1.145–1.149)	0.953 (0.950–0.957)	1.084 (1.077–1.091)
Female sex	1.137 (1.114–1.161)	1.137 (1.092–1.185)	1.140 (1.061–1.226)
65–74 years old	0.767 (0.751–0.784)	0.664 (0.640–0.688)	0.645 (0.605–0.688)
75–84 years old	0.543 (0.531–0.554)	0.359 (0.346–0.373)	0.310 (0.289–0.333)
≥85 years old	0.321 (0.311–0.330)	0.075 (0.068–0.082)	0.049 (0.040–0.0605)
CCI 2	1.205 (1.183–1.228)	1.171 (1.131–1.213)	1.242 (1.165–1.324)
CCI ≥3	1.078 (1.057–1.098)	1.255 (1.210–1.301)	1.145 (1.071–1.224)
Pneumonia	0.520 (0.508–0.533)	1.425 (1.377–1.474)	1.082 (1.013–1.156)

NIV: non-invasive ventilation. IMV: invasive ventilation. CCI: Charlson comorbidity index.

## Supplementary Materials

The following are available online at https://www.mdpi.com/2077-0383/8/10/1621/s1, Table S1: Readmission rates and discharge location of admissions for exacerbation of COPD from 2001 to 2015 in Spain in according to form of ventilation. Table S2: Discharge units for exacerbation of COPD from 2001 to 2015 in Spain according to form of ventilation.
