# Supplementary material for: Trends in the Use and Outcomes of Mechanical Ventilation among Patients Hospitalized with Acute Exacerbations of COPD in Spain, 2001 to 2015
Source: J Clin Med. 2019 Oct 4;8(10):1621. doi: 10.3390/jcm8101621 (PMC6832372; doi:10.3390/jcm8101621)
Supplement: Supplementary file 1 [file jcm-08-01621-s001.pdf]

Supplementary table 1. Readmission rates and discharge location of admissions for exacerbation of COPD from 2001 to 2015 in Spain in according to form of ventilation.

	Form of ventilation	2001/03	2004/06	2007/09	2010/12	2013/15	Total
**READMISSIONS**	NV*	41383(15.98)	46207(16.94)	50549(17.4)	46164(17.39)	44881(17.54)	229184(17.06)
n (%)	NIV*	816(16.72)	1289(16.58)	2310(17.67)	3672(18.56)	4211(18.08)	12298(17.87)
	IMV	459(12.35)	523(13.96)	408(12.69)	262(11.17)	234(12.29)	1886(12.63)
	NIV+IMV	50(11.24)	115(13.58)	118(12.24)	130(12.48)	131(11)	544(12.12)
**DISCHARGE LOCATION**							
Home, n(%)	NV*	234665(90.63)	245569(90.03)	261017(89.83)	237327(89.38)	227507(88.9)	1206085(89.76)
Other hospital, n(%)		5041(1.95)	5630(2.06)	6459(2.22)	5506(2.07)	5137(2.01)	27773(2.07)
Nursing home, n(%)		0(0)	896(0.33)	3103(1.07)	3900(1.47)	4540(1.77)	12439(0.93)
Other, n(%)		2713(1.05)	2908(1.07)	1620(0.56)	2323(0.87)	3845(1.5)	13409(1)
Home, n(%)	NIV*	4197(85.99)	6647(85.48)	11065(84.65)	16520(83.48)	18983(81.5)	57412(83.43)
Other hospital, n(%)		90(1.84)	131(1.68)	210(1.61)	379(1.92)	546(2.34)	1356(1.97)
Nursing home, n(%)		0(0)	31(0.4)	146(1.12)	334(1.69)	521(2.24)	1032(1.5)
Other, n(%)		35(0.72)	35(0.45)	72(0.55)	181(0.91)	348(1.49)	671(0.98)
Home, n(%)	IMV*	2166(58.29)	2176(58.07)	1887(58.68)	1363(58.1)	1073(56.36)	8665(58.04)
Other hospital, n(%)		273(7.35)	260(6.94)	215(6.69)	199(8.48)	167(8.77)	1114(7.46)
Nursing home, n(%)		0(0)	24(0.64)	56(1.74)	41(1.75)	52(2.73)	173(1.16)
Other, n(%)		50(1.35)	23(0.61)	12(0.37)	9(0.38)	11(0.58)	105(0.7)
Home, n(%)	NIV+IMV*	285(64.04)	579(68.36)	601(62.34)	655(62.86)	754(63.31)	2874(64.02)
Other hospital, n(%)		22(4.94)	39(4.6)	52(5.39)	67(6.43)	90(7.56)	270(6.01)
Nursing home, n(%)		0(0)	5(0.59)	17(1.76)	26(2.5)	48(4.03)	96(2.14)
Other, n(%)		1(0.22)	6(0.71)	8(0.83)	7(0.67)	11(0.92)	33(0.74)

**p*-trend<0.05. NV: No ventilation. NIV: noninvasive ventilation. IMV: invasive ventilation.

Supplementary table 2. Discharge units for exacerbation of COPD from 2001 to 2015 in Spain according to form of ventilation.

	Type	2004/06	2007/09	2010/12	2013/15	Total
Internal medicine, n(%)	NV*	84750(45.87)	150139(51.67)	139567(52.56)	133508(52.17)	507964(50.96)
Respiratory medicine, n(%)		54253(29.36)	93733(32.26)	88791(33.44)	87784(34.3)	324561(32.56)
Geriatric medicine, n(%)		2686(1.45)	4739(1.63)	6384(2.4)	7706(3.01)	21515(2.16)
Intensive care medicine, n(%)		549(0.3)	489(0.17)	447(0.17)	393(0.15)	1878(0.19)
Other medical service, n (%)		5052(2.73)	8762(3.02)	9193(3.46)	9297(3.63)	32304(3.24)
Emergency room, n (%)		2939(1.59)	8209(2.83)	9039(3.4)	9022(3.53)	29209(2.93)
Other, n (%)		34339(18.58)	24105(8.3)	11805(4.45)	7840(3.06)	78089(7.83)
Internal medicine, n(%)	NIV*	1682(29.74)	4006(30.65)	6611(33.41)	7894(33.89)	20193(32.67)
Respiratory medicine, n(%)		3250(57.46)	8054(61.61)	11609(58.66)	13764(59.09)	36677(59.34)
Geriatric medicine, n(%)		11(0.19)	58(0.44)	244(1.23)	206(0.88)	519(0.84)
Intensive care medicine, n(%)		138(2.44)	213(1.63)	299(1.51)	359(1.54)	1009(1.63)
Other medical service, n (%)		64(1.13)	215(1.64)	425(2.15)	474(2.03)	1178(1.91)
Emergency room, n (%)		10(0.18)	57(0.44)	272(1.37)	302(1.3)	641(1.04)
Other, n (%)		494(8.73)	456(3.49)	311(1.57)	272(1.17)	1533(2.48)
Internal medicine, n(%)	IMV*	637(25.05)	959(29.82)	653(27.83)	515(27.05)	2764(27.62)
Respiratory medicine, n(%)		883(34.72)	1191(37.03)	896(38.19)	716(37.61)	3686(36.83)
Geriatric medicine, n(%)		7(0.28)	9(0.28)	12(0.51)	11(0.58)	39(0.39)
Intensive care medicine, n(%)		540(21.23)	750(23.32)	618(26.34)	534(28.05)	2442(24.4)
Other medical service, n (%)		61(2.4)	127(3.95)	111(4.73)	93(4.88)	392(3.92)
Emergency room, n (%)		6(0.24)	10(0.31)	4(0.17)	6(0.32)	26(0.26)
Other, n (%)		401(15.77)	163(5.07)	49(2.09)	27(1.42)	640(6.39)
Internal medicine, n(%)	NIV+IMV*	118(20.03)	212(21.99)	253(24.28)	299(25.1)	882(23.3)
Respiratory medicine, n(%)		324(55.01)	492(51.04)	528(50.67)	600(50.38)	1944(51.35)
Geriatric medicine, n(%)		2(0.34)	2(0.21)	7(0.67)	1(0.08)	12(0.32)
Intensive care medicine, n(%)		92(15.62)	202(20.95)	208(19.96)	241(20.24)	743(19.62)
Other medical service, n (%)		10(1.7)	14(1.45)	32(3.07)	33(2.77)	89(2.35)
Emergency room, n (%)		0(0)	0(0)	1(0.1)	2(0.17)	3(0.08)
Other, n (%)		42(7.13)	41(4.25)	11(1.06)	12(1.01)	106(2.8)

*p-trend<0.05. NV: No ventilation. NIV: noninvasive ventilation. IMV: invasive ventilation.
Data for year 2001 to 2003 was not available.
